# Fatigue and diabetes self-management among operable pancreatic cancer survivors with comorbid diabetes: comparative propensity analysis

**DOI:** 10.1007/s11764-025-01838-w

**Published:** 2025-06-04

**Authors:** Hsuan-Ju Kuo, Yun-Jen Chou, Hui-Ying Yang, Shiow-Ching Shun

**Affiliations:** 1 School of Nursing, College of Medicine, National Taiwan University, Taipei, Taiwan; 2 School of Nursing, College of Medicine, Chang Gung University, Taoyuan, Taiwan; 3 Division of Colon and Rectal Surgery, Chang Gung Memorial Hospital at Linkou, Taoyuan, Taiwan; 4 Institute of Clinical Nursing, College of Nursing, National Yang Ming Chiao Tung University, Taipei, Taiwan

**Keywords:** Diabetes, Diabetes self-management, Fatigue, Operable pancreatic cancer, Pancreatic cancer, Propensity analysis

## Abstract

**Purpose:**

Operable pancreatic cancer survivors with diabetes experience fatigue and engage less in diabetes self-management, which could lead to worse health-related outcomes. However, little is known about the impact of having both diseases on aspects of fatigue and diabetes self-management. This study aimed to compare characteristics of fatigue and diabetes self-management between operable pancreatic cancer (OPC) survivors with diabetes and people who have diabetes without OPC.

**Methods:**

This is a cross-sectional study with a total of 207 participants with diabetes (57 OPC survivors and 150 without OPC). Fatigue and diabetes self-management were measured by the Fatigue Symptom Inventory and the Summary of Diabetes Self-Care Activities, respectively. Data were matched by age, gender, and diabetes treatment using propensity analysis. We used generalized estimating equations to compare the fatigue characteristics and diabetes self-management between the two groups.

**Results:**

The final analysis included 98 participants with diabetes (49 OPC survivors and 49 without OPC) after propensity score matching. OPC survivors with diabetes perceived higher fatigue interference in general daily activities, work activities, and enjoyment of life and performed fewer diet and foot self-management compared to people who had diabetes without OPC.

**Conclusions:**

OPC survivors with diabetes perceived more fatigue interference and engaged less in healthy eating and foot care. Future longitudinal studies should focus on examining the interplay between fatigue and diabetes self-management during the perioperative period.

**Implications for Cancer Survivors:**

Future interventions addressing perceived fatigue interference and barriers to daily healthy eating and foot care are crucial for OPC survivors with diabetes.

**Supplementary Information:**

The online version contains supplementary material available at https://doi.org/10.1007/s11764-025-01838-w.

## Introduction

Cancer and diabetes are both prevalent and devastating diseases, leading to disability and death worldwide [1, 2]. Among people with cancer, individuals with pancreatic cancer have a significantly higher prevalence (68%) of coexisting diabetes than people with other common cancers [3]. Notably, about 15–60% of the individuals with operable pancreatic cancer experience deteriorated glucose metabolism during the perioperative period [4], which could lead to an increased risk for poor outcomes, such as infections and longer length of hospital stay [5]. Less performance in diabetes self-management and more symptom burdens are hypothesized as explanations for worse outcomes in cancer survivors with comorbid diabetes [6]. However, studies on cancer survivors with comorbid diabetes seldom include operable pancreatic cancer survivors, a population most likely impacted by uncontrollable diabetes and daily symptom burdens.

Among the myriad of symptoms that operable pancreatic cancer survivors experience, fatigue is highly prevalent (about 82%) [7]. Fatigue, a persistent subjective feeling of tiredness that is typically not relieved by rest, is known to impair daily function and quality of life in both people with cancer [8] and diabetes [9]. Clinical guidelines have emphasized the importance of regularly evaluating patients’ fatigue characteristics, including intensity, duration, and perceived disturbances [8]. However, fatigue remains an under-evaluated and under-treated symptom in people with cancer, including pancreatic cancer survivors [10], possibly due to its complex nature. More research on understanding which fatigue characteristics healthcare providers should pay attention to is crucial for improving fatigue management in operable pancreatic cancer survivors with comorbid diabetes.

Diabetes self-management, such as eating a healthy diet and self-monitoring of blood glucose, is essential for adequate glycemic control. However, because diabetes self-management regimens can be complicated, studies have shown that diabetes self-management typically decreases after a cancer diagnosis and its subsequent treatments [11, 12]. In turn, poor management of blood glucose levels could lead to higher cancer-related mortality in cancer survivors with diabetes [13]. However, there remains a gap in the literature regarding the state of diabetes self-management in people with both burdens of operable pancreatic cancer and diabetes, leading to limited information on diabetes self-management guideline development for operable pancreatic cancer survivors with diabetes. Given that the gold standard of diabetes care guidelines is primarily based on studies in people with type 2 diabetes [14], comparing diabetes self-management between operable pancreatic cancer survivors with diabetes and people who have type 2 diabetes without operable pancreatic cancer is the first step to understand better the current state of diabetes self-management in people living with both operable pancreatic cancer and diabetes.

One previous study has shown that higher levels of fatigue and lower performance in diabetes self-management are related to worse quality of life in operable pancreatic cancer survivors with diabetes [15]. Nonetheless, little is known about which aspect of fatigue and diabetes self-management should healthcare providers prioritize to target first. Thus, the purpose of this study was to compare fatigue characteristics and diabetes self-management between operable pancreatic cancer survivors with diabetes and people who have diabetes without operable pancreatic cancer to gain more insights into which aspects require healthcare providers’ attention to improve the care for operable pancreatic cancer survivors with diabetes.

This study was guided by the theory of unpleasant symptoms [16]. According to the theory of unpleasant symptoms, physiologic influencing factors, including the types of diseases, could impact a person’s symptom experiences and their outcome performances. Specifically, people with more comorbidities (e.g., having both operable pancreatic cancer and diabetes) could have more burdens of fatigue and perform less daily diabetes self-management. Therefore, we hypothesize that operable pancreatic cancer survivors with diabetes experience higher fatigue intensity, longer fatigue duration, and worse fatigue interference and perform less diabetes self-management than people who have diabetes without operable pancreatic cancer.

## Methods

### Design and data collection

The authors conducted a secondary data analysis combining two datasets. One dataset was from 57 operable pancreatic cancer survivors with comorbid diabetes, and the other was from 150 participants who had type 2 diabetes without operable pancreatic cancer. Both parent studies used a cross-sectional, correlational design, and the numbers of participants that were potentially eligible, confirmed eligible, and included were reported in the parent studies [15, 17].

Data collected from people with a dual diagnosis of operable pancreatic cancer and diabetes were part of a study conducted from 2016 to 2018 focusing on fatigue, diabetes self-management, and quality of life in people who were diagnosed with diabetes and had undergone pancreatic tumor surgery [15]. Data collected from people with type 2 diabetes were gathered in 2021 for testing the mediation effects of fatigue on the link between influencing factors and diabetes self-management [17]. Data for both parent studies were collected by a nurse researcher who worked as a registered nurse in a diabetes ward for 3 years and had interview skills training in the pancreatic surgical clinic for 1 year. After obtaining the participants’ written informed consent, the nurse researcher collected data using structured questionnaires via face-to-face interviews with the participants. All participants from the two parent studies completed the surveys for the key variables in this study (i.e., fatigue and diabetes self-management); the response rate was 100% for both groups.

### Study setting and sample

Both parent studies were conducted in metropolitan areas of northern Taiwan. A purposive sample of participants with both operable pancreatic cancer and diabetes was recruited from a pancreatic surgical outpatient clinic in a medical center with about 1800 medical-surgical beds and about 100 outpatient visits to the pancreatic surgical outpatient clinic per session. A convenience sample of participants who had type 2 diabetes was recruited from two diabetes outpatient clinics of a regional hospital that had about 500 medical-surgical beds and about 30–70 diabetes outpatient visits for each clinic per session. The eligibility criteria for the parent studies are listed in Table 1.

**Table 1:** Eligibility criteria of the parent studies

Dataset for participants with both operable pancreatic cancer and diabetes	Dataset for participants with type 2 diabetes
1. People who underwent pancreatic surgery for pancreatic or ampullary cancer and were diagnosed with diabetes mellitus	1. People who were diagnosed with type 2 diabetes
2. Aged 20 years or above	2. Aged 20 years or above
3. Could communicate in Chinese or Taiwanese and were willing to participate and sign the informed consent	3. Could speak or read in Chinese or Taiwanese and were willing to participate and sign the informed consent
4. People who were receiving active treatment for cancer other than pancreatic cancer, and who were diagnosed with cognitive impairment-related diseases (e.g., dementia) were excluded	4. People who were pregnant, had a fever, were having treatment for cancer, were diagnosed with chronic fatigue syndrome, or had fibromyalgia were excluded

Before performing the data analysis, we conducted a power analysis using G*Power version 3.1.9.7 to estimate the required sample size for this study. Given that we used generalized estimating equations (i.e., an extension of the generalized linear model) [18] to compare the fatigue characteristics and diabetes self-management between the two groups, we calculated the required sample size using the multiple linear regression method in the G*Power software. With alpha = 0.05, power = 80%, *R*^2^ = 0.3 [15], and entering 9 potential variables (i.e., fatigue intensity, duration, pattern, interference, diet, physical activity, foot care, taking medication, and self-monitoring of blood glucose), at least a sample size of 46 is required. Thus, this study has adequate power.

### Instruments

The authors used a structured questionnaire in Chinese to collect data. The questionnaire included a researcher-designed demographic and clinical characteristics form, the Fatigue Symptom Inventory (FSI), and the Summary of Diabetes Self-Care Activities (SDSCA).

#### Demographic and clinical characteristics

Participants self-reported their demographic data, including age, gender, years of education, and marital status. Clinical information, including body mass index (BMI), diabetes duration, HbA1c levels, insulin injections, pancreatic disease diagnosis, pancreatic surgery type, and cancer stage, was recorded from the medical records.

#### Fatigue Symptom Inventory

Participants’ fatigue characteristics were measured by the FSI. This scale comprises 14 items that evaluate fatigue intensity, interference, duration, and pattern [19]. Other than item 12 (assessing the number of days feeling fatigued in the past 7 days) and item 14 (providing qualitative information related to fatigue patterns), items of the FSI are measured on an 11-point Likert scale, with 0 meaning no fatigue at all and 10 meaning extreme fatigue. A higher total fatigue score represents a higher fatigue level. The FSI has demonstrated acceptable discriminative validity to differentiate the fatigue experiences between people with cancer and those without cancer [20]. In this study, the Cronbach’s alpha for the combined sample was 0.92.

#### Summary of Diabetes Self-Care Activities

Diabetes self-management was measured using the SDSCA. This 10-item scale evaluates the number of days for the past week that the participants followed healthy eating habits, engaged in physical activities, took prescribed medication, monitored their blood glucose levels, and performed foot care [21]. Higher scores on the SDSCA indicate higher levels of diabetes self-care performance for the past 7 days. The SDSCA demonstrates adequate reliability and validity across seven studies [21]. In the present study, the Cronbach’s alpha of the SDSCA for the combined sample was 0.68, which is consistent across different language versions of the SDSCA and reflects the multidimensional nature of diabetes self-management. Given the lower Cronbach’s alpha level, we compared the various aspects of diabetes self-management separately instead of comparing the total SDSCA score, as recommended by a previous study [17].

### Data analysis

The authors used IBM SPSS Statistics version 29.0 for data analysis, including statistical assumption checks, descriptive statistics, and propensity score analysis. Descriptive statistics, including means and standard deviations, were calculated for continuous variables, and counts and percentages were examined for categorical variables. Although no data were missing, we chose the listwise deletion option in the SPSS software during data analysis.

The authors used the propensity score matching method to reduce potential confounding effects among the two groups [22]. We performed a logistic regression analysis to estimate the propensity scores. For variable selection of the regression model, potential confounding variables were determined according to studies on people with diabetes and cancer. Specifically, potential confounding variables most frequently reported in the previous studies were considered. Age, gender, and diabetes-related medication treatment were reported to be important factors related to fatigue [9, 10] and diabetes self-management [11, 23] and were included in the model to estimate propensity scores.

Using the estimated propensity scores, we performed one-to-one matching of participants who had both operable pancreatic cancer and diabetes to those who had diabetes without operable pancreatic cancer. We applied the greedy nearest neighbor matching algorithm without replacement using a caliper width of 0.05 [22]. To check the balance of the matched pairs, graphic and numeric diagnostics, including the propensity score distribution plot, standard mean difference (SMD) and the results from the generalized estimating equations (GEE) were used. SMD greater than 0.25 [24] and the Wald chi-square test with a *p*-value less than 0.05 [22] were indicators of distribution imbalance between the matched pairs. We included variables that showed unbalanced distributions after propensity score matching in the GEE model as covariates to adjust for possible residual confounding effects [22]. The matched data were analyzed with GEE applying the exchangeable working correlation matrix, which is considered appropriate for cross-sectional data that are clustered within groups [18].

The dataset of people who had both operable pancreatic cancer and diabetes includes people with ampullary cancer because the surgical procedure and prognosis were similar to patients who were diagnosed with pancreatic cancer [25]. To ensure the robustness of the current study results, we conducted a sensitivity analysis excluding people with ampullary cancer [26]. The sensitivity analysis produced the same results as the original analysis, providing support that the present study results were robust.

Ethical considerations.

Both parent studies were approved by the institutional review board (IRB) at each study site. The study on people with both operable pancreatic cancer and diabetes was approved by the National Taiwan University Hospital IRB (identification number: 201611038RIN), and the study on people with type 2 diabetes was approved by the National Taiwan University Hospital Hsin-Chu Branch IRB (identification number: 109–132-E).

## Results

### Participant characteristics

Table 2 presents the demographic and clinical characteristics of all 207 participants and the subset of 98 participants after propensity score matching. Before propensity matching, the participants who had both operable pancreatic cancer and diabetes were mostly female (56%), married (78%), about 65 years old, diagnosed with diabetes for about 4 years, and were mostly prescribed insulin injections (54%). Participants who had diabetes without operable pancreatic cancer were mostly male (59%), were married (75%), reported a mean age of 59 years old, were diagnosed with diabetes for about 7 years, and mostly were not prescribed insulin injections (77%). The two groups were not statistically significantly different only in years of education (SMD = 0.1; *p* = 0.557) and HbA1c levels (SMD = 0; *p* = 0.969), and the rest of the demographic and clinical variables were statistically significantly different.

**Table 2:** Demographic and clinical characteristics of all participants (*n* = 207) and the propensity score-matched subset of participants (*n* = 98)^a^

Variables	All patients (*n* = 207)				Matched set (*n* = 98)			
	OPC & DM (*n* = 57)	DM (*n* = 150)	SMD	*p*^b^	OPC & DM (*n* = 49)	DM (*n* = 4*9*)	SMD	*p*^c^
Age, years (SD)	65.5 (11)	59 (13.2)	0.51	< 0.001	65.1 (10.7)	63.8 (12.2)	0.11	0.587
Education, years (SD)	10.6 (4.3)	11 (4)	0.10	0.557	11.2 (4.6)	10.4 (3.8)	0.19	0.337
Gender				0.046				0.544
Male (%)	25 (43.9)	89 (59.3)	0.15		23 (46.9)	26 (53.1)	0.06	
Female (%)	32 (56.1)	61 (40.7)	0.15		26 (53.1)	23 (46.9)	0.06	
Marital status				0.045				0.671
Married (%)	45 (78.9)	113 (75.3)	0.04		39 (79.6)	36 (73.5)	0.06	
Not married (%)	3 (5.3)	21 (14)	0.7		3 (6.1)	5 (10.2)	0.04	
Divorced (%)	1 (1.8)	8 (5.3)	0.04		1 (2.0)	4 (8.2)	0.06	
Widowed (%)	8 (14)	8 (5.3)	0.09		6 (12.2)	4 (8.2)	0.04	
BMI (SD)	21.9 (3.2)	27.2 (4.6)	1.24	< 0.001	22.1 (3.3)	25.7 (4.7)	0.89	< 0.001
DM duration, years (SD)	4.1 (6.4)	7.6 (7.1)	0.50	0.001	4.0 (6.3)	10.4 (8)	0.88	< 0.001
HbA1 C (SD)	7.3 (1.4)	7.3 (1.3)	0	0.969	7.3 (1.4)	7.5 (1.4)	0.14	0.420
Insulin injections				< 0.001				0.685
No (%)	26 (45.6)	116 (77.3)	0.32		24 (49)	22 (44.9)	0.04	
Yes (%)	31 (54.4)	34 (22.7)	0.32		25 (51)	27 (55.1)	0.04	
Diagnosis				–				–
PDAC (%)	37 (64.9)	–	–		32 (65.3)	–	–	
AC (%)	9 (15.7)	–	–		9 (18.4)	–	–	
PNET (%)	6 (10.5)	–	–		5 (10.2)	–	–	
IPMC (%)	5 (8.9)	–	–		3 (6.1)	–	–	
Surgery type				–				–
PPPD (%)	24 (42.1)	–	–		21 (42.9)	–	–	
TP (%)	21 (36.8)	–	–		17 (34.7)	–	–	
PD (%)	12 (21.1)	–	–		11 (22.4)	–	–	
Cancer stage				–				–
I (%)	12 (21)	–	–		11 (22.4)	–	–	
II (%)	41 (72)	–	–		34 (69.4)	–	–	
III (%)	2 (3.5)	–	–		2 (4.1)	–	–	
IV (%)	2 (3.5)	–	–		2 (4.1)	–	–	

–: not applicable

*OPC* operable pancreatic cancer; *DM* diabetes mellitus; *SMD* standard mean difference, raw differences were calculated for proportions; *BMI* body mass index; *DM* diabetes mellitus; *PDAC* pancreatic ductal adenocarcinoma; *AC* ampullary cancer; *PNET* pancreatic neuroendocrine tumor; *IPMC* intraductal papillary mucinous cancer; *PPPD* pylorus-preserving pancreaticoduodenectomy; *TP* total pancreatectomy; *PD* pancreaticoduodenectomy

^a^Data are presented as mean (standard deviation) or number (%)

^b^*p*-values were examined by independent *t*-test or chi-square test/Fisher’s exact test to compare the two groups

^c^*p*-values were examined by generalized estimating equation based on exchangeable working correlation to compare the two groups

After the propensity score matching process, the demographic and clinical variables of the two groups were not statistically significantly different (i.e., SMD = 0.04 ~ 0.19; *p*-value = 0.337 ~ 0.685) except for body mass index (SMD = 0.89; *p* < 0.001) and diabetes duration (SMD = 0.88; *p* < 0.001). Specifically, participants who had both operable pancreatic cancer and diabetes had lower body mass index and shorter diabetes duration than the group without cancer. To adjust for residual confounding effects, body mass index and diabetes duration were entered into the GEE models for comparison of fatigue and diabetes self-management between the two groups. Of particular note, the sample with both operable pancreatic cancer and diabetes showed consistent disease characteristics before and after propensity score matching. That is, most of the participants had pancreatic ductal adenocarcinoma, underwent pylorus-preserving pancreaticoduodenectomy, and were diagnosed with stage two cancer, indicating the matched subset was representative of the original dataset.

### Comparing fatigue characteristics among the matched pairs

Table 3 shows the results for comparing fatigue intensity, duration, pattern, and interference between participants who had both operable pancreatic cancer and diabetes and those who had diabetes without operable pancreatic cancer. The group with both disease burdens had statistically significantly higher levels of overall perceived fatigue interference (coefficient = 0.73, *p* = 0.046), especially in general daily activities (coefficient = 1.41, *p* = 0.007), work activities (coefficient = 1.31, *p* = 0.011), and enjoyment of life (coefficient = 0.86, *p* = 0.049) than the group who had diabetes without operable pancreatic cancer.

**Table 3:** Matched-pair analysis of fatigue characteristics after propensity score matching

Variables	OPC & DM (*n* = 49)		DM (*n* = 49)		Coefficient (95% Wald confidence interval)^a^	*p*-value^a^
	Mean	SD	Mean	SD		
Fatigue intensity						
Worst fatigue	3	3.04	3.08	2.72	− 0.12 (− 1.45, 1.22)	0.866
Average fatigue	1.73	1.77	1.78	1.78	− 0.06 (− 0.83, 0.72)	0.882
Fatigue duration						
Number of days fatigued	2.78	2.9	3.86	2.71	− 0.90 (− 2.18, 0.38)	0.168
How much of the day fatigued	2.35	2.91	2.47	2.57	0.11 (− 1.22, 1.44)	0.871
Daily fatigue pattern					0.11 (− 0.72, 0.93)	0.799
No fatigue, *n* (%)	20	− 40.8	16	− 32.7		
Worse in the morning, *n* (%)	3	− 6.1	5	− 10.2		
Worse in the afternoon, *n* (%)	6	− 12.2	10	− 20.4		
Worse in the evening, *n* (%)	3	− 6.1	7	− 14.3		
No consistent daily pattern, *n* (%)	17	− 34.7	11	− 22.4		
Perceived fatigue interference	1.32	1.96	0.76	1.65	0.73 (0.01, 1.44)	0.046
General daily activities	2.14	3.01	0.86	2.1	1.41 (0.38, 2.43)	0.007
Ability to bathe and dress	0.63	2.08	0.41	1.74	0.48 (− 0.29, 1.25)	0.222
Work activity	1.82	3.09	0.82	2.16	1.31 (0.30, 2.31)	0.011
Ability to concentrate	1	2.18	0.96	1.87	0.19 (− 0.63, 1.00)	0.656
Relations with others	0.92	2.16	0.47	1.6	0.50 (− 0.20, 1.20)	0.165
Enjoyment of life	1.47	2.52	0.69	1.98	0.86 (0.01, 1.71)	0.049
Mood	1.24	2.27	1.08	2.38	0.36 (− 0.54, 1.27)	0.431
Total fatigue score	20.43	24.12	17.23	20.5	4.60 (− 4.66, 13.87)	0.33

Note: Scores were measured using the Fatigue Symptom Inventory, with each item ranging from 0 to 10, except for item 12 (number of days fatigued, ranging from 0 to 7) and item 14 (daily fatigue pattern: no fatigue, worse in the morning, worse in the afternoon, worse in the evening, or no consistent daily pattern). The total fatigue score was calculated by adding items 1 to 13, with a possible range from 0 to 127. Higher scores represented worse fatigue

*OPC* operable pancreatic cancer, *DM* diabetes mellitus

^a^Coefficient, 95% Wald confidence interval, and *p*-value were examined by generalized estimating equation based on exchangeable working correlation with adjustment of BMI and diabetes duration to compare the two groups; the reference group was the participants who had DM without OPC

Participants living with both operable pancreatic cancer and diabetes had a higher total fatigue score (mean = 20.4, SD = 24.1) than participants who had diabetes without operable pancreatic cancer (mean = 17.2, SD = 20.5); however, we observed no statistically significant difference between the two groups (coefficient = 4.6, *p* = 0.330). No statistically significant differences were found in fatigue intensity, duration, and pattern between the two groups. Notably, although the difference in the number of days feeling fatigued (coefficient =  − 0.90, *p* = 0.168) between the two groups was not statistically significant, the magnitude of the difference was larger than the difference in perceived fatigue interference (coefficient = 0.73, *p* = 0.046). The magnitude of the difference in the number of days fatigued could represent clinical significance, indicating that participants with both disease burdens tend to report fewer days of fatigue for the past week than those without cancer.

### Comparing diabetes self-management among the matched pairs

Table 4 shows the results of comparing diabetes self-management for the past week between the two groups. Operable pancreatic cancer survivors with diabetes followed a less healthy diet (coefficient =  − 2.14, *p* < 0.001) and performed less foot care (coefficient =  − 2.13, *p* = 0.004) for the past week than people who had diabetes without operable pancreatic cancer. Other diabetes self-management domains, including engaging in physical activity, taking medication, and self-monitoring of blood glucose, were comparable among the two groups. Additionally, taking medications regularly was the top-ranked diabetes self-care activity for both groups. Foot care was ranked the least performed self-care activity for the group with both operable pancreatic cancer and diabetes.

**Table 4:** Matched-pair analysis of diabetes self-management after propensity score matching

Summary of Diabetes Self-Care Activities	OPC & DM (*n* = 49)	SD	DM (*n* = 49)	SD	Coefficient (95% Wald confidence interval)^a^	*p*-value^a^
	Mean		Mean			
Diet	3.05	1.37	5.48	1.79	− 2.14 (− 2.99, − 1.29)	< 0.001
Physical activity	3.94	2.86	3.44	2.07	0.33 (− 0.86, 1.53)	0.585
Foot care	2.29	3.18	4.95	2.67	− 2.13 (− 3.57, − 0.70)	0.004
Medication	6.65	1.07	6.28	1.56	0.57 (− 0.24, 1.39)	0.167
Self-monitoring of blood glucose	3.82	3.36	3.35	2.91	0.34 (− 1.21, 1.89)	0.668

Note: Scores were measured using the Summary of Diabetes Self-Care Activities, with each item ranging from 0 to 7

*OPC* operable pancreatic cancer, *DM* diabetes mellitus

^a^Coefficient, 95% Wald confidence interval, and *p*-value were examined by generalized estimating equation based on exchangeable working correlation with adjustment of BMI and diabetes duration to compare the two groups; the reference group was the participants who had DM without OPC

## Discussion

Operable pancreatic cancer survivors with diabetes are likely to experience higher fatigue levels and engage less in diabetes self-management; nonetheless, the extent to which both disease burdens affect fatigue and diabetes self-management is unknown. This comparison study using propensity score analysis filled in this knowledge gap by revealing that adults who had concurrent operable pancreatic cancer and diabetes experienced higher levels of perceived fatigue interference and performed less diet and foot self-management compared to people who had diabetes without operable pancreatic cancer.

In the present study, operable pancreatic cancer survivors with comorbid diabetes reported a mean worse fatigue intensity score of 3, an average of 2.78 days fatigued per week, and a mean fatigue interference score of 1.32. Our participants reported lower scores across all fatigue characteristics compared to previous studies measuring fatigue using the FSI in people with cancer (mainly breast cancer survivors, fatigue intensity scores of 4.58 ~ 5.25 [27], a mean of 4.5 days fatigued per week [28], and fatigue interference scores of 3.38 ~ 4.79 [27]), and the scores reported in this study are similar to those reported in healthy adults (mean fatigue intensity of 2.8, a mean of 3.1 days fatigued per week, and a mean fatigue interference score of 1.3 [29]). Nonetheless, a fatigue intensity score of 3 has reached a clinically meaningful cut-off because this severity is associated with more fatigue interference and duration [30]. In terms of the lower fatigue scores in our study, given that pancreatic cancer is known to be a challenging diagnosis with less likelihood of survival and higher chances of recurrence [31], patients may be more reluctant to report fatigue to their healthcare providers with fear that the reporting of this symptom may hinder their cancer treatment [32], especially when treatments are perceived as their only choice and chance of survival [33]. Moreover, we conducted this study in Taiwan, where the Confucian philosophical root is deeply planted in Taiwanese culture, and reporting a nonvisible symptom such as fatigue, whether due to cancer or diabetes, could represent shame and weakness [32, 34], which could potentially lead to the under-reporting of the fatigue scores. Further qualitative studies exploring the barriers to reporting fatigue, especially under the influence of cancer mortality and different cultural backgrounds, are needed to provide more insights into the patients’ perspectives.

Our study revealed that operable pancreatic cancer survivors with diabetes experienced worse fatigue interference than those who had diabetes without cancer. Notably, both groups had comparable fatigue intensity, duration, and patterns. One possible explanation for this finding could be that operable pancreatic cancer survivors with diabetes are experiencing an additive effect from both diseases [35], impacting their perception of fatigue interference. Specifically, perceived fatigue interference can be influenced by the emotional burdens of having cancer. For example, a recent meta-analysis showed that fear of cancer recurrence is associated with fatigue [36]. As a result, although operable pancreatic cancer survivors with diabetes had similar severity of fatigue intensity and duration to those who had diabetes without operable pancreatic cancer, adding on the emotional burdens of having cancer, fatigue is more disturbing to their daily activities, work, and enjoyment of life. This finding highlights the importance of assessing and managing fatigue interference for operable pancreatic cancer survivors with diabetes. Furthermore, understanding the effects of having the psychological burden of both diseases on fatigue interference is a critical next step. Future studies should focus on investigating the modifiable factors (e.g., depressive symptoms and sleep quality) related to perceived fatigue interference. Researchers should also test the effectiveness of interventions targeting fatigue interference, especially disturbances in daily activities, work, and enjoyment of life.

Individuals with both operable pancreatic cancer and diabetes reported spending fewer days following a healthy diet compared to those who had diabetes without operable pancreatic cancer. This finding is consistent with previous studies in people with both cancer and diabetes, suggesting that cancer has a negative impact on diabetes diet management [37, 38]. Particularly, one study has shown that in cancer survivors with comorbid diabetes, more negative beliefs about cancer and higher levels of cancer-related symptoms are associated with decreased diabetes diet adherence [37]. Given that pancreatic cancer is known to be a life-threatening disease, cancer survivors could naturally prioritize cancer-related treatments before diabetes diet management. Further research on how to tailor a healthy eating plan for operable pancreatic cancer survivors with diabetes while considering the burden and complexity of having both diseases is needed. Future studies are advised to explore possible barriers (e.g., multiple symptom burdens) and facilitators (e.g., social support) to maintaining a healthy diet for operable pancreatic cancer survivors with diabetes to improve their health-related outcomes. Longitudinal studies are also needed to examine the interplay between cancer surgery recovery and healthy eating related to glycemic control.

This study showed that operable pancreatic cancer survivors with diabetes performed less foot care for the past week compared to people who had diabetes without cancer. Compared to other diabetes self-care activities (e.g., monitoring blood glucose levels and taking medications) that are often directly linked to glycemic control and mortality, foot care is related to the prevention of complications such as foot ulcers, which could be the reason why it was often overlooked [39]. For cancer survivors with diabetes, cancer could be perceived as a more immediate threat to survival than diabetes [40], resulting in less awareness of diabetic foot prevention. Moreover, compared to people with diabetes without cancer, cancer survivors with diabetes had less access to diabetes knowledge and less self-efficacy in diabetes self-management [41], which could also lead to patients’ lower foot care performance.

Diabetic foot prevention and foot care are essential for cancer survivors with diabetes because about 34% of people with diabetes will have a chance of developing foot ulcers during their lifetime, and people with diabetic foot ulcers are more at risk for depression, mortality, and worse quality of life [42]. Foot self-care, such as daily foot inspection and foot hygiene, should optimally be practiced every day [14, 39]. However, our study revealed that operable pancreatic cancer survivors with diabetes only performed foot care for about 2 days/week. Our findings regarding the disparity in foot self-management in operable pancreatic cancer survivors with diabetes warrant further research on why patients perform less foot care and how to intervene to assist patients in integrating more foot care into their daily lives.

### Strengths and limitations

The strength of this study is examining the impact of having both operable pancreatic cancer and diabetes on fatigue and diabetes self-management while incorporating a comparison group of people who had diabetes without operable pancreatic cancer, providing more insights into specific aspects of fatigue and diabetes self-management that require more attention. Of particular note, a major strength of this study is the attempt to control for confounding effects using propensity score analysis. However, propensity score matching has its limitations, such as the inability to control for unmeasured confounders (e.g., marital status or partnership, annual income, or socioeconomic status) and loss of sample size during the matching process [22]. Another limitation that needs to be considered is the cross-sectional design of the study, which only captured how having both operable pancreatic cancer and diabetes impacted fatigue and diabetes self-management at a given point in time. Specifically, operable pancreatic cancer survivors with diabetes could have different fatigue trajectories and challenges in performing diabetes self-management throughout the perioperative period. Therefore, future studies using a longitudinal design to understand how patients are performing diabetes self-management and what the barriers are during different time points will provide valuable information on how and when to intervene. Another limitation is that, although the FSI is a measure with good psychometric properties [20], it may not capture all nuances of fatigue experiences unique to individuals with both cancer and diabetes. Future qualitative studies are required to explore differences in fatigue experience in operable pancreatic cancer survivors with diabetes. Lastly, we collected data in metropolitan areas of Taiwan, and the study results might not be generalizable to patients in rural settings or settings outside Taiwan. Nonetheless, the study provides valuable insights into the concurrent impact of operable pancreatic cancer and diabetes on fatigue and diabetes self-management that could be informative for healthcare providers caring for operable pancreatic cancer survivors with diabetes.

## Conclusion

This study revealed that operable pancreatic cancer survivors living with diabetes perceived more fatigue interference in general daily activities, work activities, and enjoyment of life and engaged less in healthy eating and foot care compared with people who have diabetes without operable pancreatic cancer. These findings indicate a need for further research on fatigue interference, healthy diet, and foot care in adults living with both operable pancreatic cancer and diabetes. Importantly, healthcare providers should pay extra attention to these aspects of fatigue and diabetes self-management when caring for operable pancreatic cancer survivors with diabetes.

## Supplementary Information

Below is the link to the electronic supplementary material.

ESM 1 (DOCX 49.0 KB)

## Data Availability

Data will be available from the corresponding author upon reasonable request.
